# Complement C5 inhibitor as a rescue therapy for acute NMOSD attacks: a multicenter retrospective cohort study in China

**DOI:** 10.3389/fimmu.2026.1917016

**Published:** 2026-09-15

**Authors:** Tao Wu, Decai Tian, Dehui Huang, Lei Wu, Jin Zhen, Jian Yin, Jia Guo, Jingli Jiang, Fan Li, Haiqiang Jin, Yiming Zheng, Zhaoxia Wang, Ran Liu, Feng Gao

**Affiliations:** 1 Department of Neurology, Peking University First Hospital, Beijing, China; 2 Department of Neurology, China National Clinical Research Center for Neurological Diseases, Beijing Tiantan Hospital, Capital Medical University, Beijing, China; 3 Department of Neurology, The First Medical Center of Chinese PLA General Hospital, Beijing, China; 4 Department of Neurology, Inner Mongolia People’s Hospital, Hohhot, Inner Mongolia, China; 5 Department of Neurology, Beijing Hospital, National Center of Gerontology, Institute of Geriatric Medicine, Chinese Academy of Medical Sciences, Beijing, China; 6 Department of Neurology, The Second Hospital of Lanzhou University, Lanzhou, China; 7 Department of Neurology, Beijing Renhe Hospital, Beijing, China

**Keywords:** acute attack, complement inhibition, eculizumab, Neuromyelitis Optica Spectrum Disorder (NMOSD), rescue therapy

## Abstract

**Background:**

Complement activation plays a key role in the pathogenesis of neuromyelitis optica spectrum disorder (NMOSD). While eculizumab effectively prevents relapses, its role in acute attacks remains uncertain.

**Methods:**

We retrospectively analyzed 38 patients with acute NMOSD treated with eculizumab across seven tertiary hospitals in China. Neurological assessments included EDSS, visual, motor, and sensory deficits, evaluated at baseline and weekly through 8 weeks. Clinical improvement was defined using prespecified criteria. Safety and adverse events were recorded.

**Results:**

Patients were predominantly female (92.1%) with a mean age of 47.5 ± 14 years. Median EDSS decreased from 4 (IQR 3–6.88) to 3 (IQR 2–6) at the 4–8-week follow-up assessment (p = 5.45 × 10^-6^), with improvement observed in at least one neurological domain in 35/38 patients (92.1%). Improvement was more frequent in visual (83.3%) and motor function (88.8%) than in sensory function (31.6%). The median time to first improvement was 2 weeks, with cumulative probabilities of 47.4%, 55.3%, and 71.1% at weeks 1, 2, and 4, respectively. In exploratory analyses, the ON+TM phenotype was associated with a lower likelihood of improvement after adjustment for age and baseline EDSS. Four patients experienced mild adverse events, and one patient with a baseline EDSS of 9 died from septic shock.

**Conclusion:**

This exploratory study observed neurological improvement following eculizumab initiation in patients with NMOSD attacks, but serious infections can occur in severely disabled patients, highlighting the need for careful monitoring and patient selection.

## 1 Introduction

Neuromyelitis Optica Spectrum Disorder (NMOSD) is a severe autoimmune inflammatory disease of the central nervous system that predominantly affects the optic nerves and spinal cord (1, 2). Current treatments for acute attacks mainly include high-dose intravenous methylprednisolone (IVMP), plasma exchange (PE), and immunoadsorption (IA) (3–5). However, complete remission after standard therapy remains limited (6), and a substantial proportion of patients respond poorly or not at all. In addition, the availability of plasma exchange and immunoadsorption is often restricted by equipment and specialized personnel requirements, highlighting the need for alternative therapeutic strategies.

Complement activation plays a central role in the pathogenesis of NMOSD attacks. Eculizumab, a monoclonal antibody targeting complement component C5 (7), has demonstrated efficacy in preventing disease relapses. Because eculizumab can achieve rapid terminal complement inhibition after infusion (8), it may also have therapeutic potential during acute attacks. However, evidence supporting its use in this setting remains limited. Limited case reports and small-scale studies have indicated such potential efficacy (9–11). Recent evidence from Rommer et al. (12) suggests that complement inhibition may be beneficial in acute NMOSD attacks. However, the early temporal dynamics of clinical improvement, potential heterogeneity in treatment response, and safety in real-world acute settings remain incompletely defined.

In this multicenter retrospective study, we analyzed 38 patients with acute NMOSD from seven tertiary centers in China who received eculizumab. We aimed to further characterize early treatment response, identify predictors of improvement, and provide additional safety data.

## 2 Methods

### 2.1 Study design and participants

This multicenter retrospective study included patients with acute NMOSD from seven tertiary hospitals in China between January 2024 and October 2025. Patients were considered eligible when they presented with a new or worsening neurological episode consistent with an NMOSD relapse, with persistent or progressive deficits despite initial acute treatment, prompting escalation to rescue therapy. No patients were excluded based on the interval from attack onset to eculizumab initiation, in order to capture the real-world use of eculizumab as rescue therapy as comprehensively as possible. All patients fulfilled the 2015 International Panel for NMO Diagnosis criteria (13) and were positive for serum AQP4-IgG detected by a fixed cell-based assay (CBA) at the local laboratory of each participating center according to routine clinical practice at the participating centers. All patients were also tested for serum MOG-IgG, which was negative in all cases. Adults (≥18 years) who received at least two doses of Eculizumab after an attack and had available follow-up data were eligible for inclusion. All consecutive patients meeting the predefined eligibility criteria during the study period were included. No eligible patients were excluded after screening. The interval from relapse onset to eculizumab initiation was recorded for all patients and was considered in the analysis. No patients included in this cohort overlapped with previously published cohorts evaluating eculizumab treatment in acute NMOSD attacks.

### 2.2 Treatment

Eculizumab was administered intravenously at 900 mg weekly for four doses as induction therapy, followed by 1200 mg every two weeks when continued. For exploratory analyses, treatment duration was categorized as <4 weeks (2–3 induction doses), 4 weeks (four induction doses), or 8 weeks (continuation beyond the induction phase through at least week 8), with the latter not necessarily representing long-term maintenance treatment. Most patients had previously received high-dose intravenous methylprednisolone (IVMP), with some additionally treated with PE or IA before eculizumab initiation due to insufficient response or disease progression. Meningococcal vaccination or short-term antibiotic prophylaxis was provided prior to treatment.

### 2.3 Clinical assessment and outcomes

Clinical data were collected from electronic medical records across participating centers. Neurological assessments were performed at baseline (before eculizumab initiation) and during follow-up within eight weeks after treatment initiation. The post-treatment EDSS value was defined as the latest available assessment between weeks 4 and 8 after eculizumab initiation. Evaluations included Expanded Disability Status Scale (EDSS) scores, visual acuity, motor strength (Medical Research Council scale), sensory function, and patient-reported symptom changes.

The primary outcome was neurological improvement following treatment, assessed by changes in EDSS scores and predefined functional recovery in the affected systems (visual, motor, or sensory; see **Supplementary Table 2**). EDSS was used to assess global disability, while domain-specific assessments were used to capture recovery within affected neurological systems. These measures were considered complementary. Secondary outcomes included the proportion of patients with clinical improvement, time to improvement, and treatment safety.

### 2.4 Statistical analysis

Continuous variables were expressed as mean ± standard deviation or median (interquartile range), and categorical variables as counts (percentages). Changes in EDSS scores were evaluated using the paired Wilcoxon signed-rank test. Time to EDSS improvement was analyzed using Kaplan–Meier methods. The event was defined as the first documented EDSS improvement after eculizumab initiation. Patients without documented EDSS improvement were censored at their last available EDSS assessment. Factors associated with clinical improvement were explored using Firth logistic regression. A two-sided P value < 0.05 was considered statistically significant. Statistical analyses were performed using R version 4.4.1.

## 3 Results

### 3.1 Baseline characteristics

A total of 38 patients were included, with a predominance of females (92.1%) and a mean age of 47.5 ± 14 years. Most patients (65.8%) had a disease duration of less than one year. Optic neuritis (ON) occurred in 39.5% of patients, transverse myelitis (TM) in 31.6%, and concurrent ON and TM in 23.7%. Overall, optic nerve involvement was observed in 63.2% of cases.

Nineteen patients were newly diagnosed and had not received prior maintenance immunotherapy, while the remainder 19 patients had relapsing disease with a median of three prior relapses. Among relapsing patients, 15(78.9%) had received prior maintenance immunotherapy.

The primary reason for initiating eculizumab was inadequate response to high-dose intravenous methylprednisolone (81.6%). Most patients (71.1%) started treatment within one month of relapse onset, with a median initiation time of 19 days. All received weekly induction therapy (900 mg), most commonly for four weeks, with 34.2% continuing standard maintenance dosing (1200 mg every two weeks). Details are provided in **Supplementary Table 1**.

### 3.2 Domain-specific neurological improvement and EDSS changes

Following treatment with eculizumab, 35 of 38 patients (92.1%) demonstrated improvement in at least one predefined neurological domain. Among patients with optic nerve involvement, 20 of 24 (83.3%) met the predefined criteria for visual improvement, including six patients who recovered from light perception to counting fingers, three who recovered from blindness to light perception or counting fingers, and the remainder who showed a measurable improvement in best-corrected visual acuity (BCVA). Four patients showed no visual improvement. Among those with motor deficits due to myelitis, 16 of 18 (88.8%) showed motor improvement. Among those with sensory deficits due to myelitis, 6 of 19 (31.6%) showed sensory improvement. Urinary retention, pruritus, and neuropathic pain also improved in several cases. Details are provided in **Supplementary Table 2**.

The median EDSS score improved from 4 (IQR, 3–6.88) at baseline to 3 (IQR 2–6) at the 4–8-week follow-up assessment. The median change in EDSS was −1 (IQR −1.75 to −0.12), indicating a significant reduction in neurological disability after eculizumab initiation (p = 5.45 × 10^-6^, see **Figures 1a, b**). Ten patients (26.3%) experienced good recovery, defined as a decrease of ≥2 EDSS points from baseline (ΔEDSS ≤ −2).

**Figure 1:**
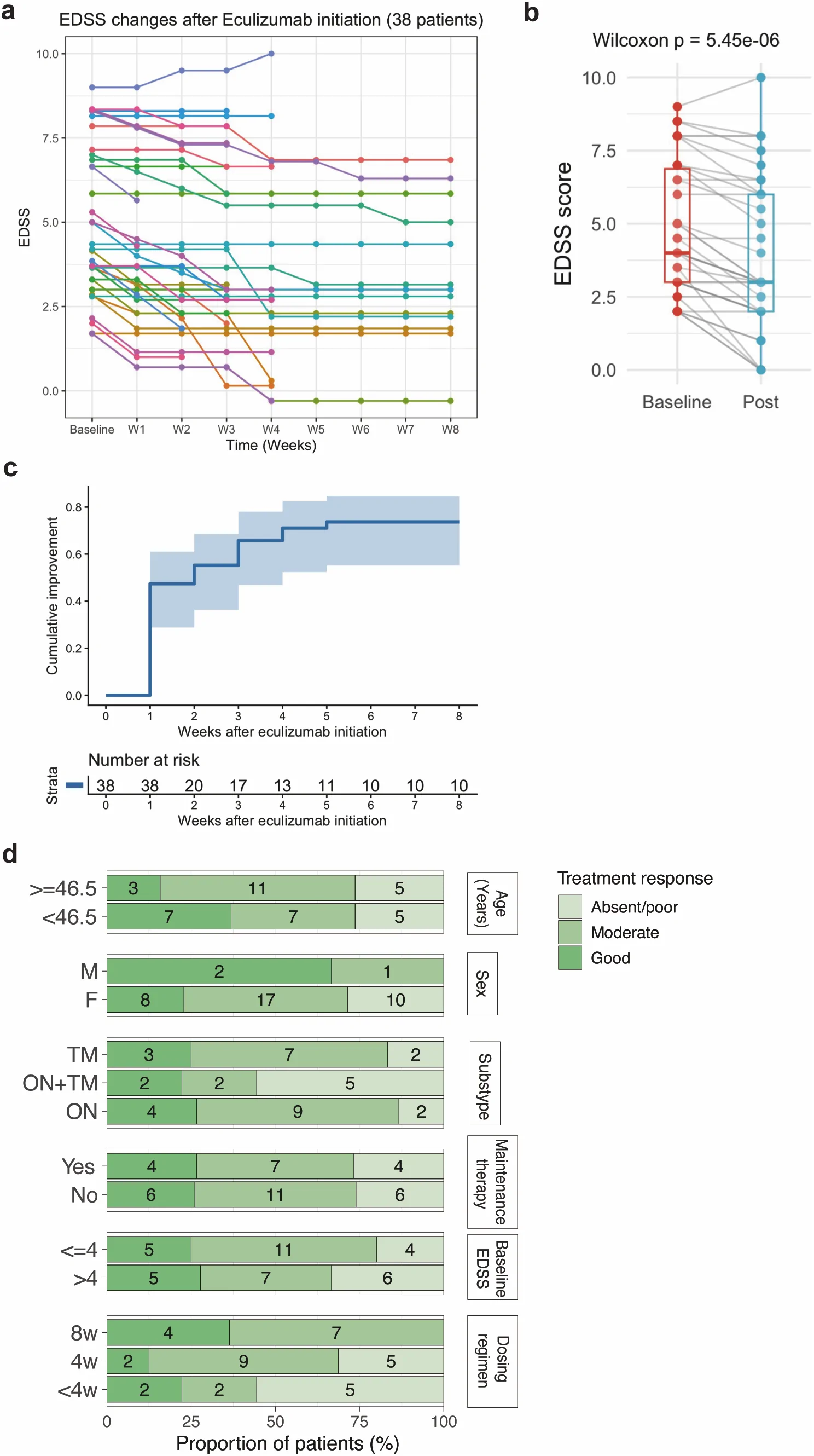
**(a)** Changes in EDSS scores following eculizumab initiation. **(b)** Distribution of baseline and 4–8-week follow-up EDSS scores. **(c)** Kaplan–Meier analysis of the cumulative probability of domain-specific clinical improvement. **(d)** Treatment response stratified by clinical and therapeutic factors. In **(c)**, Domain-specific clinical improvement was assessed separately using predefined criteria for visual, motor, and sensory recovery. In **(d)**, treatment response was defined by ΔEDSS as absent/poor (ΔEDSS ≥ 0), moderate (−2 < ΔEDSS < 0), or good recovery (ΔEDSS ≤ −2).

### 3.3 Time to EDSS improvement

The median time to first documented clinical improvement after initiation of eculizumab was 2 weeks (95% CI 1–4 weeks). Kaplan–Meier analysis showed that the earliest improvement occurred within the first week. The cumulative probability of improvement at weeks 1, 2, and 4 was 47.4%, 55.3%, and 71.1%, respectively (**Figure 1c**). These findings indicate that clinical improvement frequently occurred within the first few weeks after treatment initiation.

### 3.4 Factors associated with clinical improvement

Through stratified descriptive analysis, ON+TM showed a higher proportion of absent/poor responses compared with ON and TM (5/9 [55.6%] vs 2/15 [13.3%] and 2/12 [16.7%], respectively). The 8-week regimen showed a higher proportion of moderate/good responses compared with 4-week and <4-week regimens (11/11 [100%] vs 11/16 [68.8%] and 4/9 [44.4%], respectively; see **Figure 1d**). In logistic regression analysis, after adjusting for age and baseline EDSS score, the ON+TM phenotype was independently associated with a lower probability of clinical improvement (OR 0.19, p = 0.033). The 8-week dosing regimen was associated with a higher likelihood of improvement (p = 0.012, **Table 1**); however, this exploratory finding should be interpreted cautiously given the limited sample size. Age, baseline EDSS score, time to eculizumab initiation, and maintenance therapy were not significantly associated with clinical improvement.

**Table 1:** Exploratory regression analysis of factors associated with clinical improvement.

Variable	OR (95% CI)	p value	OR* (95% CI)	p value*
Age	1.01 (0.96–1.06)	0.706	1.02 (0.97–1.08)	0.511
Baseline EDSS score	0.89 (0.64–1.21)	0.443	0.85 (0.59–1.19)	0.352
ON+TM phenotype	0.18 (0.04–0.85)	0.030	0.19 (0.04–0.87)	0.033
8-week dosing regimen	13.8 (1.50–1839)	0.016	14.8 (1.61–1969)	0.012
Maintenance therapy	0.95 (0.23–4.12)	0.942	0.92 (0.23–3.93)	0.908
Number of prior attacks	0.99 (0.89–1.15)	0.825	1.01 (0.91–1.17)	0.834
Time of eculizumab initiation	1.02 (0.98–1.07)	0.409	1.01 (0.98–1.07)	0.502

*Adjusted for age and baseline EDSS score.

OR indicates odds ratio; CI, confidence interval; EDSS, Expanded Disability Status Scale.

To explore potential clinical heterogeneity, treatment outcomes were descriptively analyzed according to prior acute therapies. Among patients who received eculizumab after IVMP alone, 22/23 (95.7%) demonstrated clinical improvement, compared with 6/6 (100%) among those who had previously received PE/IA and 1/3 (33.3%) among those who received IVMP combined with IVIg.

Clinical improvement was also observed across patients with different background maintenance immunotherapy strategies. Among patients without maintenance immunotherapy, 20/23 (87.0%) demonstrated clinical improvement. Among patients receiving maintenance immunotherapy, improvement was observed across different treatment classes, including B-cell depletion therapies (6/6, 100%), IL-6 receptor antagonists (1/1, 100%), BLyS/APRIL inhibition (1/1, 100%), and antimetabolites (3/3, 100%). Given the small sample sizes within each subgroup, these findings were interpreted descriptively and considered exploratory.

### 3.5 Safety

Four patients (10.5%) experienced mild adverse events, including urinary tract infection (n = 2) and skin rash (n = 2). One patient with severe neurological disability at baseline (EDSS = 9) developed septic shock and subsequently died (detailed in **Supplementary Table 3**). No cases of meningococcal infection were observed. No hypertension, headache, diarrhea, nausea, vomiting, anemia, or thrombocytopenia were reported during treatment.

## 4 Discussion

In this multicenter study, eculizumab was associated with a high rate of neurological improvement in patients with acute NMOSD. More than 90% of patients demonstrated clinical improvement, accompanied by a significant reduction in EDSS scores at eight weeks. Improvement was observed across multiple neurological domains, with higher rates of visual and motor recovery, whereas sensory improvement was less pronounced.

Complement-mediated astrocyte injury is a key mechanism in NMOSD pathogenesis. By inhibiting complement component C5, eculizumab can rapidly suppress terminal complement activation (8), providing a biological rationale for its use in the acute setting. Furthermore, recent preclinical evidence suggests that C5 blockade can rapidly attenuate astrocyte destruction and promote remyelination, supporting a potential role for complement inhibition in limiting acute tissue injury and facilitating recovery (14). Consistent with this mechanism, we observed that clinical improvement frequently occurred within the first few weeks after treatment initiation. Our exploratory analyses further suggested heterogeneity in treatment response. Patients with combined optic neuritis and transverse myelitis (ON+TM) were less likely to improve, however, further studies using standardized domain-specific outcome measures are needed to validate this observation. Treatment duration showed an exploratory association with outcome; however, this relationship may reflect treatment selection and post-treatment clinical evolution rather than a causal effect.

These findings complement prior evidence from Rommer et al. (12) suggesting a potential role for complement inhibition in acute NMOSD attacks. Our study provides additional real-world data from a multicenter Chinese cohort and further characterizes clinical outcomes following eculizumab initiation, including the timing of neurological improvement, domain-specific functional recovery, and safety considerations. The lack of a significant association between treatment timing and outcome may be related to the heterogeneous treatment window, limited sample size, and potential confounding by clinical factors influencing treatment initiation. Therefore, whether earlier complement inhibition provides superior recovery remains to be determined. Notably, one fatal septic event occurred in a patient with severe baseline disability after eculizumab initiation. However, the patient developed invasive pulmonary aspergillosis rather than meningococcal infection, and the event occurred in the context of severe baseline impairment and immobility. Although a contribution from complement inhibition cannot be excluded, the fatal outcome was likely multifactorial, highlighting the need for careful patient selection and close monitoring.

Several limitations should be acknowledged. First, the retrospective single-arm design without a control group limits causal inference. Because most patients received prior corticosteroids and some additionally underwent PE, IA, IVIg, or continued maintenance immunotherapy, the observed improvement cannot be attributed solely to eculizumab and may also reflect delayed effects of previous therapies or spontaneous recovery. In addition, the timing of eculizumab initiation was heterogeneous (median, 19 days after attack onset), and some patients treated later may have entered the subacute recovery phase rather than during active inflammatory worsening. Furthermore, not all patients received PLEX/IA before eculizumab initiation, reflecting real-world treatment decisions influenced by clinical circumstances and treatment accessibility. Therefore, this study was not designed to compare eculizumab with established rescue therapies, and its incremental benefit remains uncertain.

Second, exploratory analyses of factors associated with improvement should be interpreted cautiously due to the small sample size, limited number of non-responders, and potential model instability. Functional outcomes were retrospectively assessed without blinded evaluation, and subjective assessment bias cannot be excluded. The requirement for at least two doses of eculizumab may also have introduced selection bias by excluding patients with early treatment discontinuation. Finally, the generalizability of our findings is limited because the cohort mainly included AQP4-IgG-positive patients from tertiary centers in China with inadequate response to initial therapy. Acute-phase eculizumab remains an exploratory approach, and prospective controlled studies are required to define its role in NMOSD attack management.

## 5 Conclusion

This exploratory study observed neurological improvement following eculizumab initiation in acute NMOSD, although a causal treatment effect cannot be established. Careful patient selection is needed due to potential infection risk in severely disabled patients. Prospective controlled studies are needed to further evaluate the role of eculizumab in acute NMOSD attacks.

## Data Availability

The original contributions presented in the study are included in the article/**Supplementary Material**. Further inquiries can be directed to the corresponding authors.
