# Supplementary material for: Complement C5 inhibitor as a rescue therapy for acute NMOSD attacks: a multicenter retrospective cohort study in China
Source: Front Immunol. 2026 Sep 15;17:1917016. doi: 10.3389/fimmu.2026.1917016 (PMC13619596; doi:10.3389/fimmu.2026.1917016)
Supplement: Supplementary file 1 [file Table1.docx]

**Supplementary Table 1.** Demographic and clinical characteristics of patients treated with Eculizumab

| Patient No. | Sex | Age group, years | Disease duration | Attack type | Clinical manifestations | No. of previous attacks | Previous maintenance therapy | Comorbidities | Serum AQP4-IgG titer* | Reason for initiating Eculizumab |
| --- | --- | --- | --- | --- | --- | --- | --- | --- | --- | --- |
| 1 | F | 70-75 | 9 months | TM | Bilateral leg weakness/numbness, bladder and bowel dysfunction | 1 | rituximab | SPS, MCTD, SS, HT, T1DM | Positive | IVMP Intolerance (post-RTX B-cell depletion, severe DM) |
| 2 | M | 40-45 | 3 months | ON | Visual loss | 0 | - | PTB history | 1:40 | IVMP Failure/Inefficacy |
| 3 | F | 40-45 | 1 month | ON+TM | Limb weakness/numbness, visual loss | 0 | - | MG | 1:40 | IVMP Failure/Inefficacy |
| 4 | F | 45-50 | 9 months | ON+TM+APS | Hiccups, limb numbness, blurred vision, diplopia, dysuria | 1 | prednisone | LC, GFAP-Ab+ | Positive | IVMP+PE Failure/Inefficacy |
| 5 | F | 60-65 | 10 years | ON+TM | Visual loss, limb weakness/numbness, chest pain | 3 | prednisone + mycophenolate mofetil | severe OP, fracture | 1:20 | IVMP Intolerance (severe OP, fracture) |
| 6 | F | 50-55 | 1.5 years | ON | Visual loss | 3 | - | OP, Dry eye | Positive | IVMP Failure/Inefficacy |
| 7 | F | 20-25 | 6 days | ON+TM+BS | Peripheral facial palsy, gaze palsy, bladder/bowel dysfunction, visual loss | 0 | - | AE, severe drug rash, DRESS, prior PCP infection, Teratoma, Oral candidiasis | 1:40 | IVMP+IVIg  Failure/Inefficacy |
| 8 | F | 65-70 | 1 month | ON+APS | Dizziness, vomiting, visual loss | 0 | - | HTN, DM, CI, Compression fracture | Positive | IVMP Failure/Inefficacy |
| 9 | F | 65-70 | 13 days | ON | Bilateral visual loss | 0 | - | CAD, RA | 1:10 | IVMP Intolerance (angina pectoris) |
| 10 | F | 60-65 | 14 years | ON | Visual loss | 3 | prednisone | None | Positive | Previous IVMP Failure/Inefficacy |
| 11 | F | 45-50 | 6 years | ON+TM | Limb numbness/weakness, visual loss | 1 | - | BC History | 1:100 | IVMP Failure/Inefficacy |
| 12 | F | 40-45 | 17 days | ON | Visual loss | 0 | - | Hyperthyroidism | 1:32 | IVMP Failure/Inefficacy |
| 13 | F | 60-65 | 14 years | ON+TM | Limb numbness/weakness, visual loss | 10 | Satralizumab | None | 1:1000 | IVMP Failure/Inefficacy |
| 14 | F | 50-55 | 21 days | ON | Visual loss | 0 | - | None | 1:1000 | IVMP Failure/Inefficacy |
| 15 | F | 45-50 | 6 years | ON | Visual loss | 4 | prednisone | None | 1:1000 | IVMP Failure/Inefficacy |
| 16 | F | 40-45 | 27 days | TM | Limb numbness/weakness | 0 | - | None | 1:100 | IVMP Failure/Inefficacy |
| 17 | F | 55-60 | 12 months | TM | Flank pain, bilateral leg numbness/weakness, bladder/bowel dysfunction | 1 | inebilizumab | OP | 1:100 | IVMP+PE Failure/Inefficacy |
| 18 | F | 35-40 | 20 days | TM | Neck pain, limb numbness/weakness | 0 | - | None | 1:1000 | IVMP Failure/Inefficacy |
| 19 | F | 30-35 | 2 months | APS+BS | Nausea/vomiting, limb numbness/weakness | 0 | - | None | 1:32 | IVMP Failure/Inefficacy |
| 20 | F | 35-40 | 8 months | TM | Posterior neck pain, limb numbness/weakness, bladder/bowel dysfunction | 3 | rituximab | AVNF | 1:32 | Previous IVMP Failure/Inefficacy  Current PE+IA Failure |
| 21 | F | 40-45 | 9 years | ON | Right eye visual loss | 11 | - | HL | 1:100 | IVMP+PE  Failure/Inefficacy |
| 22 | F | 30-35 | 2 years | CE | Left limb weakness | 2 | mycophenolate mofetil | SLE, SS | 1:100 | IVMP Failure/Inefficacy |
| 23 | F | 50-55 | 1 week | TM | Bilateral leg weakness/numbness | 0 | - | None | 1:100 | Worsening despite IVMP |
| 24 | F | 35-40 | 2 weeks | TM | Bilateral leg weakness/numbness | 0 | - | SS | 1:3200 | Worsening despite IVMP |
| 25 | F | 30-35 | 2 weeks | ON | Acute right eye visual loss | 0 | - | HT | 1:32 | Worsening despite IVMP |
| 26 | M | 35-40 | 14 days | ON | Bilateral visual loss | 0 | - | None | 1:20 | Worsening despite IVMP+IVIg |
| 27 | F | 60-65 | 50 days | ON+TM | Bilateral blindness, quadriparesis | 0 | - | None | 1:320 | IVMP+IVIg Failure/Inefficacy |
| 28 | F | 70-75 | 2 weeks | TM | Bilateral leg weakness/numbness, bladder/bowel dysfunction | 0 | - | CAG, AF, CI | 1:320 | Worsening despite IVMP |
| 29 | M | 65-70 | 6 months | TM+BS | Dizziness, Limb numbness/weakness | 0 | - | HTN | 1:10 | IVMP Failure/Inefficacy |
| 30 | F | 30-35 | 2 years | ON | Left eye visual loss | 3 | - | None | 1:320 | IVMP Failure/Inefficacy |
| 31 | F | 45-50 | 20 years | ON+TM | Bilateral visual loss, Limb weakness | 30 | inebilizumab | None | Positive | IVMP Failure/Inefficacy |
| 32 | F | 20-25 | 1 months | ON | Right eye visual loss | 1 | eculizumab | None | 1:1000 | Previous IVMP Failure/Inefficacy |
| 33 | F | 20-25 | 15 days | ON | Right eye pain/visual loss | 1+ | mycophenolate mofetil | None | 1:100 | IVMP+PE Failure/Inefficacy |
| 34 | F | 35-40 | 18 years | ON | Bilateral visual loss | 8 | rituximab | None | 1:100 | IVMP+PE Failure/Inefficacy |
| 35 | F | 50-55 | 3 months | ON+TM | Left eye visual loss, Limb numbness/weakness | 0 | - | None | 1:320 | IVMP Failure/Inefficacy |
| 36 | F | 60-65 | 14 years | TM | Bilateral leg weakness/numbness, | 6 | telitacicept | OP | 1:40 | Worsening despite IVMP |
| 37 | F | 55-60 | 1 months | TM+APS | Nausea/vomiting, limb numbness | 0 | - | HTN, fracture | 1:100 | Worsening despite IVMP |
| 38 | F | 50-55 | 23 years | TM+BS | Limb numbness/weakness | 5 | inebilizumab | HTN | 1:320 | Previous IVMP Failure/Inefficacy |

*AQP4-IgG results were extracted from the original medical records. Where available, antibody titers are presented; otherwise, qualitative seropositivity ("Positive") was recorded.

**Abbreviations:** TM: Transverse myelitis; ON: Optic neuritis; BON: Bilateral optic neuritis; BS: Brainstem syndrome; APS: Area postrema syndrome; CE: Cerebral syndrome; AE, autoimmune encephalitis; AVNF, avascular necrosis of femoral head; BC, breast cancer; CAD, coronary artery disease; CI, cerebral infarction; DM, diabetes mellitus; DRESS, drug reaction with eosinophilia and systemic symptoms; GFAP-Ab+, GFAP antibody positive; HL, hyperlipidemia; HT, Hashimoto's thyroiditis; HTN, hypertension; LC, lung cancer; MCTD, mixed connective tissue disease; MG, myasthenia gravis; OP, osteoporosis; RA, rheumatoid arthritis; SLE, systemic lupus erythematosus; SPS, stiff-person syndrome; SS, Sjögren's syndrome; T1DM, type 1 diabetes mellitus; PTB, Pulmonary tuberculosis; IVMP: Intravenous methylprednisolone; PE: Plasma exchange; IA: Immunoadsorption; IVIg: Intravenous immunoglobulin.
