# Supplementary material for: Complement C5 inhibitor as a rescue therapy for acute NMOSD attacks: a multicenter retrospective cohort study in China
Source: Front Immunol. 2026 Sep 15;17:1917016. doi: 10.3389/fimmu.2026.1917016 (PMC13619596; doi:10.3389/fimmu.2026.1917016)
Supplement: Supplementary file 2 [file Table2.docx]

**Supplementary Table 2.** Treatment regimen and outcomes of patients receiving Eculizumab

| Patient No. | Sex | Age group, years | This onset to IVMP (days) | Other acute therapies | This onset to Ecu (days) | Eculizumab regimen | EDSS before Ecu | EDSS after Ecu (W4-8) | Visual improvement* | Motor improvement* | Sensory improvement* | Adverse Events |
| --- | --- | --- | --- | --- | --- | --- | --- | --- | --- | --- | --- | --- |
| 1 | F | 70-75 | Not used | - | 8 | 900mg qw *4w→1200mg q2w | 8 | 7 | - | Y | Y | Urinary Tract Infection |
| 2 | M | 40-45 | 7 | IVMP | 90 | 900mg qw *4w | 3 | 2 | Y | - | - | None |
| 3 | F | 40-45 | 31 ± | IVMP | 37 | 900mg qw *4w | 3.5 | 0 | Y | Y | Y | None |
| 4 | F | 45-50 | 7 | IVMP+PE | 42 | 900mg qw *4w→1200mg q2w | 2.5 | 0 | - | Y | - | None |
| 5 | F | 60-65 | Not used | - | 14 | 900mg qw *2w | 2 | 2 | N | - | Y | None |
| 6 | F | 50-55 | 10 ± | IVMP | 14 | 900mg qw *4w→1200mg q2w | 3 | 2 | Y | - | - | None |
| 7 | F | 20-25 | 3 ± | IVMP+IVIg | 19 | 900mg qw*2w | 3 | 3 | N | - | - | Skin rash |
| 8 | F | 65-70 | 27 | IVMP | 33 | 900mg qw *3w | 4 | 3 | Y | - | - | None |
| 9 | F | 65-70 | 13 | IVMP (discontinued due to intolerance) | 16 | 900mg qw *4w | 3 | 2 | Y | - | - | None |
| 10 | F | 60-65 | Not used | - | 20 | 900mg qw *2w | 2 | 0 | Y | - | - | None |
| 11 | F | 45-50 | 3 | IVMP | 3 | 900mg qw *4w | 6 | 6 | Y | N | N | None |
| 12 | F | 40-45 | 8 | IVMP | 17 | 900mg qw *4w | 3 | 3 | Y | - | - | None |
| 13 | F | 60-65 | 5 | IVMP | 6 | 900mg qw *4w | 6.5 | 6.5 | Y | - | - | None |
| 14 | F | 50-55 | 14 | IVMP | 21 | 900mg qw *4w | 3 | 2 | Y | - | - | None |
| 15 | F | 45-50 | 1 | IVMP | 15 | 900mg qw *4w | 4 | 3 | Y | - | - | None |
| 16 | F | 40-45 | 21 | IVMP | 30 | 900mg qw *4w | 7 | 6 | - | Y | N | Urinary Tract Infection |
| 17 | F | 55-60 | 10 | IVMP+PE | 49 | 900mg qw *4w→1200mg | 7 | 5 | - | Y | N | None |
| 18 | F | 35-40 | 10 | IVMP | 38 | 900mg qw *4w | 3.5 | 3 | - | Y | N | None |
| 19 | F | 30-35 | Time unknown | IVMP | 50 | 900mg qw *4w | 2.5 | 2.5 | - | - | N | None |
| 20 | F | 35-40 | Not used | PE+IA | 31 | 900mg qw *4w→1200mg q2w*2w | 4.5 | 2.5 | - | Y | N | None |
| 21 | F | 40-45 | 2 | IVMP+PE | 25 | 900mg qw *4w | 4.5 | 4.5 | Y | - | - | None |
| 22 | F | 30-35 | 4 | IVMP | 27 | 900mg qw *4w | 5 | 3 | - | Y | N | None |
| 23 | F | 50-55 | 2 | IVMP | 7 | 900mg qw *2w | 8 | 8 | - | Y | N | None |
| 24 | F | 35-40 | 1 | IVMP | 7 | 900mg qw *2w | 8 | 8 | - | Y | N | None |
| 25 | F | 30-35 | 1 | IVMP | 3 | 900mg qw *4w | 4 | 3 | Y | - | - | None |
| 26 | M | 35-40 | 5 | IVMP+IVIg+Satralizumab | 15 | 900mg qw *2w | 4 | 2 | Y | - | - | None |
| 27 | F | 60-65 | 3 and 50 | IVMP+IVIg | 50 | 900mg qw *2w | 9 | 9.5 | N | N | - | Severe Infection, Death |
| 28 | F | 70-75 | 2 | IVMP | 8 | 900mg qw *4w→1200mg q2w | 6.5 | 5.5 | - | Y | Y | None |
| 29 | M | 65-70 | 13 | IVMP | 11 | 900mg qw *4w→1200mg q2w | 8 | 7.5 | - | Y | N | None |
| 30 | F | 30-35 | 3 | IVMP | 9 | 900mg qw *4w→1200mg q2w | 2 | 0 | Y | - | - | None |
| 31 | F | 45-50 | 0 | IVMP | 3 | 900mg qw *4w→1200mg q2w | 8.5 | 7.5 | N | Y | N | None |
| 32 | F | 20-25 | Not used | - | 19 | 900mg qw *4w→1200mg q2w | 5 | 3 | Y | - | - | None |
| 33 | F | 20-25 | 15 | IVMP+PE | 30 | 900mg qw *4w→1200mg q2w | 2 | 1 | Y | - | - | None |
| 34 | F | 35-40 | 2 | IVMP+PE | 16 | 900mg qw *4w→1200mg q2w | 5 | 4 | Y | - | N | None |
| 35 | F | 50-55 | 81 | IVMP | 90 | 900mg qw *4w | 4 | 3 | Y | Y | Y | Skin rash |
| 36 | F | 60-65 | 1 | IVMP | 15 | 900mg qw *4w | 8.5 | 8 | - | Y | Y | None |
| 37 | F | 55-60 | 16 | IVMP+ inebilizumab | 40 | 900mg qw *2w | 2 | 1 | Y | - | N | None |
| 38 | F | 50-55 | Not used | - | 30 | 900mg qw *4w→1200mg q2w | 7 | 6.5 | - | Y | - | None |

* Predefined Criteria for Domain-Specific Clinical Improvement: Visual improvement: ≥1 level gain in visual acuity or increased BCVA; Motor improvement: ≥1 point gain in MRC score in any affected limb; Sensory improvement: ≥2-segment descent of sensory level or resolution of deficit. “Y” indicates improvement; “N” no improvement; “–” no related symptoms.

**Abbreviations:** IVMP: Intravenous methylprednisolone; PE: Plasma exchange; IA: Immunoadsorption; IVIg: Intravenous immunoglobulin.
