# Supplementary material for: Complement C5 inhibitor as a rescue therapy for acute NMOSD attacks: a multicenter retrospective cohort study in China
Source: Front Immunol. 2026 Sep 15;17:1917016. doi: 10.3389/fimmu.2026.1917016 (PMC13619596; doi:10.3389/fimmu.2026.1917016)
Supplement: Supplementary file 3 [file Table3.docx]

**Supplementary Table 3.** Clinical characteristics of the patient with fatal infection after eculizumab treatment (Patient 27).

| **Item** | **Description** |
| --- | --- |
| Age group/Sex | 60-65/F |
| Attack phenotype | ON + TM |
| Neurological deficits | Bilateral blindness; quadriparesis |
| MRI findings | C4–T2 spinal cord lesion |
| AQP4-IgG | 1:320 |
| Previous therapy | IVMP (day 3 and day 50), IVIg (day 4 and day 50), no PE |
| Eculizumab initiation | Day 50 after onset |
| Eculizumab exposure | 900 mg weekly ×2 doses |
| Prophylaxis | Meropenem; no meningococcal vaccination |
| Time from first dose to death | 24 days |
| Infection | Severe pneumonia; invasive pulmonary aspergillosis |
| Organism | Non-encapsulated organism; no meningococcal infection |
| Cause of death | Septic shock |
| Autopsy | Not performed |
